# Neuroticism, perceived stress, adverse life events and self-efficacy as predictors of the development of functional somatic disorders: longitudinal population-based study (DanFunD)

**DOI:** 10.1192/bjo.2023.644

**Published:** 2024-01-25

**Authors:** Marie Weinreich Petersen, Tina Birgitte Wisbech Carstensen, Kaare Bro Wellnitz, Eva Ørnbøl, Lisbeth Frostholm, Thomas Meinertz Dantoft, Torben Jørgensen, Lene Falgaard Eplov, Per Fink

**Affiliations:** Research Clinic for Functional Disorders and Psychosomatics, Aarhus University Hospital, Denmark; and Department of Clinical Medicine, University of Aarhus, Denmark; Center for Clinical Research and Prevention, Bispebjerg/Frederiksberg Hospital, Frederiksberg, Denmark; Center for Clinical Research and Prevention, Bispebjerg/Frederiksberg Hospital, Frederiksberg, Denmark; and Department of Public Health, Faculty of Medical Sciences, Copenhagen University, Denmark; Copenhagen Research Center for Mental Health – CORE, Mental Health Center Copenhagen, Denmark

**Keywords:** Epidemiology, functional somatic syndromes, neuroticism, stress, functional somatic disorder

## Abstract

**Background:**

Functional somatic disorder (FSD) is a unifying diagnosis that includes functional somatic syndromes such as irritable bowel, chronic widespread pain (CWP) and chronic fatigue. Several psychological factors are associated with FSD. However, longitudinal population-based studies elucidating the causal relationship are scarce.

**Aims:**

To explore if neuroticism, perceived stress, adverse life events (ALEs) and self-efficacy can predict the development of FSD over a 5-year period.

**Method:**

A total of 4288 individuals who participated in the DanFunD baseline and 5-year follow-up investigations were included. FSD was established at both baseline and follow-up, with symptom questionnaires and diagnostic interviews. Neuroticism was measured with the short-form NEO Personality Inventory, perceived stress with the Cohen's Perceived Stress Scale, ALEs with the Danish version of the Cumulative Lifetime Adversity Measure and self-efficacy with the General Self-Efficacy Scale. Associations were investigated with multiple logistic regression models.

**Results:**

Perceived stress predicted incident FSD, irritable bowel, CWP and chronic fatigue (odds ratios: 1.04–1.17). Neuroticism predicted incident FSD and chronic fatigue (odds ratios: 1.03–1.16). ALEs predicted incident FSD, CWP and chronic fatigue (odds ratios: 1.06–1.18). An increase in perceived stress from baseline to follow-up was associated with incident FSD, irritable bowel, CWP and chronic fatigue (odds ratios: 1.05–1.22). Contrary, an increase in self-efficacy seemed to be a protective factor (odds ratios: 0.89–0.99).

**Conclusions:**

High neuroticism, high perceived stress and a high number of ALEs are risk factors for the development of FSD. Particularly perceived stress seems to be an important contributor to the onset of FSD.

Functional somatic disorder (FSD) is a unifying diagnosis that includes a range of functional somatic syndromes (FSS) such as irritable bowel syndrome, fibromyalgia and chronic fatigue syndrome, and it can be conceptualised as the diagnostic concept bodily distress syndrome.^1^ It is characterised by persisting patterns of physical symptoms that cannot be better explained by other physical or mental conditions.^1^ FSD may be severely disabling and cause great emotional distress for the patients, and severe cases have an excessive use of healthcare services (e.g. repeated hospital admissions and medical investigations, and fruitless treatment attempts).^1^ The resulting costs are high for both patients and society in terms of healthcare and work-related costs.

## Aetiology of functional somatic disorders

FSD is considered to have a multifactorial aetiology involving both biological, social and psychological factors. However, psychological and social factors have shown to be rather underresearched compared with biological factors.^2^ Previous research has shown that having high levels of neuroticism ^3,4^ and stress,^5–7^ a high incidence of trauma/adverse life events (ALEs)^8,9^ and low levels of self-efficacy^10^ may be important contributors to the onset of FSD.

So far, most studies investigating the importance of psychological illness mechanisms for the development of FSD have been carried out in selected patient samples and cross-sectional studies, and there is a lack of general population-based studies based on large, randomly obtained samples. In two recent population-based studies, we have investigated the associations between FSD and neuroticism, perceived stress, accumulated number of ALEs and self-efficacy, respectively.^11,12^ However, these studies were cross-sectional, thereby not providing insight into the potential causal relationship.

## Objectives

The present study included longitudinal data from two consecutive investigations of the same general population cohort. Based on our previous studies, it was hypothesised that higher levels of neuroticism, perceived stress and number of ALEs would predict the development of new cases of FSD over a 5-year period, whereas the influence of self-efficacy would be less important. The objective was twofold. First, to investigate if neuroticism, perceived stress, ALEs and self-efficacy could predict the development of incident FSD over a 5-year time period. Second, under the assumption that neuroticism was stable during the 5-year period, to investigate if a change in perceived stress, number of ALEs and self-efficacy from baseline to follow-up would influence the development of incident FSD at follow-up.

## Method

### Study participants

For the current study, two investigations from the Danish Study of Functional Disorders (DanFunD) were included: data from the DanFunD baseline part two cohort were gathered in the years 2012–2015 and data for the 5-year follow-up cohort were gathered in the years 2018–2020.^13^ An overview of the study design and measurement points is displayed in Supplementary Fig. 1 available at https://doi.org/10.1192/bjo.2023.644. For the baseline investigation, participants were randomly drawn from the Danish Civil Registration System. The exclusion criteria were not being born in Denmark, not being a Danish citizen and pregnancy.

A total of 7493 (29.5% of the invited participants) men and women were included in the DanFunD baseline part two cohort. Age ranged from 18 to 72 years, all participants were born in Denmark and lived in the western part of greater Copenhagen. Participants completed questionnaires about physical symptoms and psychological factors, lifestyle, etc. A stratified subsample (*n* = 2450) of all participants with high symptom scores on the DanFunD baseline symptom questionnaires were invited to participate in a diagnostic interview (the Research Interview for Functional Somatic Disorders (RIFD)), together with every tenth baseline participant.^14^ Of the 2450 invited participants, 1590 (64.9%) accepted and participated in the interview.

For the DanFunD 5-year follow-up investigation, 7289 participants from the DanFunD part two baseline cohort were invited and 4288 (58.8%) participated. Of them, 1452 were invited to participate in another RIFD interview; 1092 (75.2%) accepted. For the follow-up investigation, the participants completed the same questionnaires as in the baseline investigation, except for measures about personality.

The study sample included in the current study comprised the participants who participated in both the DanFunD baseline and follow-up investigations (4288 completed the questionnaires and 1092 completed the diagnostic interviews). The flow of study participants is displayed in Supplementary Fig. 2.

### Ethics statement

The authors assert that all procedures contributing to this work comply with the ethical standards of the relevant national and institutional committees on human experimentation and with the Helsinki Declaration of 1975, as revised in 2008. All procedures involving human patients were approved by the Ethical Committee of the Capital Region (approval numbers H-3-2011-081 and H-3-2012-015), and all participants gave written informed consent.

The current study was part of a pre-registration on ClinicalTrials.gov (identifier NCT05631860). A few additions to the original protocol was made: self-efficacy was included as a primary independent variable to be investigated alongside neuroticism, perceived stress and ALEs. Analyses investigating the role of changes in perceived stress, ALEs and self-efficacy were added.

### Dependent variables

#### FSDs

The unifying diagnostic construct of bodily distress syndrome was used as the primary operationalisation of FSD. It divides patients into two subgroups: a single-organ subgroup (i.e. individuals with symptoms from one or two of four symptom clusters: cardiopulmonary, gastrointestinal, musculoskeletal and general symptoms/fatigue) and a multi-organ subgroup (i.e. individuals with symptoms from at least three of the four symptom clusters).^15,16^ The construct is well-validated and has been shown to encompass a range of FSS.^17^ In this paper, the FSD diagnosis is conceptualised as bodily distress syndrome, but we also include analyses on three well-known FSS: irritable bowel,^18^ chronic widespread pain (CWP)^19^ and chronic fatigue.^20^

#### Assessment of FSDs

The assessment of FSD was conducted both at baseline and follow-up. Cases with FSD (single- and multi-organ type) were identified by the self-reported Bodily Distress Syndrome Checklist,^15^ assessing physical symptoms that had been bothering the participant within the past 12 months. Additionally, a stratified subsample of participants with a clinical diagnosis of FSD was identified with a diagnostic interview, which was developed to be used as a second-phase tool after a respondent's self-reported symptoms in questionnaires.^14^ The diagnostic interviews were performed by trained primary care physicians over the telephone. During the interview, physicians assessed whether a symptom pattern was caused by an FSD or if it was caused by another physical or mental condition. The diagnostic interview has shown good criterion validity for identifying individuals with FSD.^14^

Individuals fulfilling the criteria for irritable bowel,^18^ CWP^19^ and chronic fatigue^20^ were identified with self-reported validated symptom questionnaires including bothersome symptoms within the past 12 months.

### Primary independent variables

#### Neuroticism

Neuroticism was measured at baseline with the Danish version of the short-form NEO Personality Inventory (NEO-PI-Rsf), which has been validated in a large sample from the Danish population and shown acceptable psychometric properties in terms of internal consistency and reliability.^21,22^ The NEO-PI-Rsf measures five domains of personality: neuroticism, extraversion, openness, agreeableness and conscientiousness. It includes 60 self-descriptive statements such as ‘I often worry about things’, which are rated with a five-point rating scale from ‘strongly disagree’ to ‘strongly agree’. In the present study, the domain for neuroticism was scored from 0 to 48, with a higher score indicating higher levels of neuroticism.

#### Perceived stress

Perceived stress was measured at both baseline and follow-up with Cohen's Perceived Stress Scale, which assesses the extent to which an individual finds their life to be unpredictable, uncontrollable and overloaded.^23^ The Danish consensus version of the scale has been shown to have good psychometric properties in terms of agreement, reliability, validity, responsiveness and interpretability.^24^ The scale consists of ten items. An example of an item could be: ‘How often have you felt that you were unable to control the important things in your life?’. Each item is rated on a five-point rating scale from ‘never’ to ‘very often’. A sum score ranging from 0 to 40 is calculated, with a higher score indicating higher levels of perceived stress.

#### ALEs

ALEs were measured both at baseline and follow-up as accumulated number of ALEs, using the Danish version of the Cumulative Lifetime Adversity Measure (CLAM), which is a well-validated measure with high construct validity and good content validity for use in population-based samples.^25^CLAM examines the cumulative effect of a number of ALEs, and takes into account the number of exposures to the same event. CLAM obtains exposure to lifetime adversity by asking the respondents whether they have experienced 37 different ALEs; however, at follow-up, the participants were asked to only rate ALEs that had occurred in the 5-year period between baseline and follow-up. CLAM includes ALEs from the following seven life categories: own illness or injury, loved one's illness or injury, violence, bereavement, social/environmental stress, relationship stress and disaster. An example of an item could be ‘Have you ever been discriminated against your ethnicity, religious background, or sexual orientation?’, which is answered ‘yes’ or ‘no’. CLAM also gives the possibility to add one other unnamed ALE. The respondents write the age at which each event occurred or an age interval if the event had occurred for a time period. A sum score ranging from 0 to 133 is calculated by counting age time points and age ranges (i.e. an age range counted for one event and an age time point counted for one event). Each type of ALE can maximally receive a score of four (i.e. the event could happen up to four times).

#### Self-efficacy

Self-efficacy was measured both at baseline and follow-up with the General Self-Efficacy Scale, which has been shown to have high internal consistency, reliability, stability and construct validity.^26^ It assesses an individual's beliefs in their own capability to perform a specific action required to attain a desired outcome. The scale consists of ten items and an example of an item could be ‘I am confident that I could deal efficiently with unexpected events’. Each item is rated on a four-point rating scale from ‘not at all true’ to ‘exactly true’. A sum score ranging from 0 to 30 is calculated, and higher scores indicate a higher degree of self-efficacy.

#### Covariates

Covariates included gender, age and subjective social status, and they were obtained by participants’ self-report at baseline. Subjective social status was measured with a single item asking the participants to rate their own social status on a scale from 1 to 10, with 1 being the lowest and 10 being the highest status in society.^27^

### Data analysis

All analyses were performed in Stata version 17.0 for Windows (StataCorp, College Station, USA).^28^ Descriptive statistics were presented as medians and interquartile ranges (IQRs) because of the non-normal distribution of the continuous variables. Categorical variables were presented as frequencies with percentages. Responders and non-responders for the 5-year follow-up investigation were compared with Wilcoxon-Mann-Whitney *U*-tests for continuous outcomes, and chi-squared tests for dichotomous outcomes.

To investigate if neuroticism, perceived stress, ALEs and self-efficacy could predict the development of incident FSD over a 5-year time period, we used multiple logistic regression models including newly developed/incident FSD (FSD negative at baseline but positive at follow-up) as a dichotomous dependent variable and neuroticism, perceived stress, number of ALEs and self-efficacy measured at baseline as primary independent median-centred continuous variables. For each of the dependent variables, all four primary independent variables were incorporated in the same regression model. The reference group comprised individuals with baseline median values of neuroticism, perceived stress, accumulated number of ALEs and self-efficacy who did not have FSD at both baseline and follow-up. Potential confounders were identified with directed acyclic graphs (DAGs) constructed in the browser-based programme DAGitty version 3.0 (for the R project; see http://dagitty.net/).^29^ The analyses were adjusted for baseline levels of gender (male as reference), median value of age and median value of social status.

To investigate if a change in perceived stress, number of ALEs and self-efficacy from baseline to follow-up would influence the development of incident FSD at follow-up, we used multiple logistic regression models including incident FSD (FSD negative at baseline but positive at follow-up) as a dichotomous dependent variable, and difference between baseline and follow-up in perceived stress, number of ALEs and self-efficacy as the independent variables. Each of the primary independent variables were investigated in separate models. The reference group comprised individuals with baseline median values of neuroticism, perceived stress, accumulated number of ALEs and self-efficacy who did not have FSD at both baseline and follow-up.

For the first objective of determining predictors of FSDs, potential confounders were identified with DAGs. Each model was adjusted for baseline levels of perceived stress, number of ALEs, self-efficacy, gender (male as reference), median value of age and median value of social status. For all analyses, model fit was assessed with Hosmer–Lemeshow *χ*^2^-tests and Pearson *χ*^2^-tests, as well as the area under the receiver operating curve and the Brier mean probability score. Linearity of each independent continuous variable was checked by expanding the model with each independent variable introduced (a) on a log scale; (b) as natural cubic splines with five knots at the 5th, 27.5th, 50th, 72.5th and 95th percentiles, according to the recommendations by Harrell;^30^ and (c) as a ten-level categorical variable. Associations were reported as odds ratios with 95% confidence intervals.

## Results

### Sample characteristics

Compared with responders, non-responders for the follow-up investigation were younger and had slightly higher baseline levels of perceived stress and neuroticism, whereas the number of ALEs was lower (Table 1). Furthermore, baseline FSD was higher in non-responders than in responders.

**Table 1:** Baseline characteristics of responders and non-responders for the 5-year follow-up investigation

	Follow-up non-responders (*n* = 3001)	Follow-up responders (*n* = 4288)	*P-*value
Women, *n* (%)	1656 (55.2)	2298 (53.5)	0.094
Age, years, median (IQR)	50 (39–61)	56 (47–64)	<0.0001
Social status, median (IQR)	7 (6–7)	7 (6–8)	<0.0001
Questionnaire-based cases, *n* (%)			
Overall FSD	593 (19.8)	638 (14.9)	<0.0001
Single-organ FSD	542 (18.1)	609 (14.2)	<0.0001
Multi-organ FSD	51 (1.7)	29 (0.7)	<0.0001
Interview-based diagnoses			
Overall FSD, *n* (%)	195 (6.5)	203 (4.7)	0.009
Single-organ FSD, *n* (%)	154 (5.1)	161 (3.8)	0.028
Multi-organ FSD, *n* (%)	41 (1.4)	42 (1.0)	0.174
Irritable bowel, *n* (%)	123 (4.1)	137 (3.2)	0.021
Chronic widespread pain, *n* (%)	156 (5.2)	175 (4.1)	0.012
Chronic fatigue, *n* (%)	332 (11.1)	329 (7.7)	<0.0001
Perceived stress, median (IQR)	11 (6–15)	10 (6–14)	<0.0001
Neuroticism, median (IQR)	16 (12–22)	15 (11–20)	<0.0001
Number of ALEs, median (IQR)	5 (3–8)	6 (4–8)	<0.0001
Self-efficacy, median (IQR)	22 (18–26)	22 (18–26)	0.087

IQR, interquartile ranges; FSD, functional somatic disorder; ALEs, adverse life events.

The median age of the 4288 individuals who participated in both the DanFunD baseline and 5-year follow-up investigations was 56 years (IQR: 47–64), and 2298 (53.6%) were women. The median baseline levels were 15 (IQR: 11–20) for neuroticism, 10 (IQR: 6–14) for perceived stress, 6 (IQR: 4–8) for ALEs and 22 (IQR: 18–26) for self-efficacy (Table 1).

The median age of the subsample of 1092 participants who went through diagnostic interviews in both the DanFunD baseline and 5-year follow-up investigations was 55 years (IQR: 47–64), and 668 (61.2%) were women. The median baseline levels were 17 (IQR: 12–24) for neuroticism, 12 (IQR: 7–16) for perceived stress, 6 (IQR: 4–9) for ALEs and 21 (IQR: 17–25) for self-efficacy (Table 1).

Qualitatively, participants who developed FSD, irritable bowel, CWP or chronic fatigue from baseline to follow-up generally had higher baseline levels of neuroticism, perceived stress and ALEs, and lower levels of self-efficacy, than the reference group (Table 2). Furthermore, incident cases showed an increase in perceived stress and ALEs, and a reduction in self-efficacy, from baseline to follow-up.

**Table 2:** Characteristics of incident cases at baseline and follow-up

	Women	Age, years	Neuroticism	Stress	ALEs	Self-efficacy	Stress	ALEs	Self-efficacy
	Baseline	Baseline	Baseline	Baseline	Baseline	Baseline	Follow-up	Follow-up	Follow-up
	%	Median (IQR)	Median (IQR)	Median (IQR)	Median (IQR)	Median (IQR)	Median (IQR)	Median (IQR)	Median (IQR)
Incident cases identified with self-reported questionnaires									
Controls^a^ (*n* = 2850)	49.0	56 (47–64)	14 (10–19)	9 (5–13)	5 (4–8)	22 (19–26)	8 (5–12)	6 (4–9)	21 (19–26)
Overall FSD (*n* = 375)	60.8	55 (46–64)	18 (13–24)	12 (8–17)	6 (4–9)	21 (17–26)	14 (9–19)	7 (5–10)	20 (16–23)
Single-organ FSD (*n* = 355)	60.9	54 (46–64)	18 (13–24)	12 (8–16)	6 (4–9)	21 (17–26)	14 (9–18)	7 (5–10)	20 (16–23)
Multi-organ FSD (*n* = 20)	60.0	56 (50–62)	22.5 (18.5–27.5)	16.5 (10–18)	6 (4.5–9.5)	20 (16–25.5)	21 (17–24)	7 (5–10.5)	18 (13.5)
Irritable bowel (*n* = 108)	76.9	52 (41–57)	20 (14–26)	14 (10–18)	6 (3.5–8)	20 (16–24)	14.5 (10–18.5)	7 (5–10)	19 (15.5–22)
Chronic widespread pain (*n* = 167)	65.9	57 (51–66)	17 (13–24)	12 (8–16)	7 (4–10)	20 (17–25)	12 (8–17)	8 (5–11)	20 (16–22)
Chronic fatigue (*n* = 204)	70.1	52 (39–61.5)	20 (15–27.5)	13.5 (8–18)	6 (4–9)	20 (16–24)	16 (11–21)	8 (5–10.5)	18 (14–22)
Incident cases diagnosed with interviews									
Controls^b^ (*n* = 505)	53.1	57 (48–65)	15 (10–20)	10 (5–14)	6 (4–8)	21 (18–26)	14 (8–19)	7 (5–10)	20 (18–25)
Overall FSD (*n* = 59)	72.9	52 (43–59)	20 (16–27)	14 (10–19)	8 (5–11)	21 (17–25)	14 (8–19)	10 (6–11)	20 (14–22)
Single-organ FSD (*n* = 50)	76.0	50.5 (44–59)	21 (16–28)	14 (10–19)	7 (5–11)	21 (16–25)	14.5 (8–19)	10 (6–11)	18 (14–22)
Multi-organ FSD (*n* = 9)	55.6	56 (42–57)	17 (16–25)	18 (8–20)	8 (8–10)	23 (19–27)	13 (9–18)	10 (10–11)	21 (20–25)

ALEs, lifetime accumulated number of adverse life events; IQR, interquartile range; FSD, functional somatic disorder.

a. No FSD, irritable bowel, chronic widespread pain or chronic fatigue according to the self-reported symptom questionnaires at both baseline and follow-up.

b. No FSD diagnosis according to the diagnostic interview at both baseline and follow-up.

### Predictors of incident FSDs

Baseline levels of neuroticism, perceived stress and ALEs predicted incident FSD at follow-up, whereas the baseline level of self-efficacy did not predict any of the FSDs (Table 3). For example, for overall FSD defined by symptom questionnaires, comparing two participants differing only on one point on baseline neuroticism but having similar levels of perceived stress and self-efficacy, number of ALEs, gender, age and social status, the participant with the highest score had a 1.04 (95% CI 1.02–1.06) times higher odds of developing incident overall FSD at follow-up; a one-point increase in baseline perceived stress resulted in a 1.06 (95% CI 1.03–1.09) times higher odds of developing incident overall FSD at follow-up; and a one-point increase in the baseline accumulated number of ALEs resulted in a 1.07 (95% CI 1.04–1.10) times higher odds of developing incident overall FSD at follow-up.

**Table 3:** Odds of incident functional somatic disorders and functional somatic syndromes

	Neuroticism	Perceived stress	Adverse life events	Self-efficacy
	Odds ratio (95% CI)	Odds ratio (95% CI)	Odds ratio (95% CI)	Odds ratio (95% CI)
FSD defined by self-reported symptom questionnaires				
Overall FSD (*n* = 375)	**1.04** (**1.02–1.06)**	**1.06** (**1.03–1.09)**	**1.07** (**1.04–1.10)**	1.01 (0.99–1.04)
Single-organ FSD (*n* = 355)	**1.03** (**1.01–1.06)**	**1.06** (**1.03–1.09)**	**1.07** (**1.04–1.10)**	1.01 (0.98–1.04)
Multi-organ FSD (*n* = 20)^a^	**1.16** (**1.10–1.23)**	**1.17** (**1.10–1.25)**	1.11 (1.00–1.24)	0.95 (0.88–1.03)
FSD diagnosis established by means of diagnostic interviews				
Overall FSD (*n* = 59)^b^	1.06 (1.00–1.12)	1.07 (1.00–1.14)	**1.10** (**1.03–1.17)**	1.03 (0.97–1.09)
Single-organ FSD (*n* = 50)^b^	1.05 (1.00–1.11)	1.05 (0.99–1.12)	1.06 (0.99–1.13)	1.02 (0.96–1.08)
Multi-organ FSD (*n* = 9)^a^	1.06 (0.98–1.15)	1.09 (0.99–1.20)	**1.18** (**1.06–1.31)**	1.06 (0.93–1.21)
FSS defined by self-reported symptom questionnaires				
Irritable bowel (*n* = 108)	1.01 (0.97–1.05)	**1.06** (**1.02–1.11)**	1.03 (0.97–1.08)	0.98 (0.94–1.02)
Chronic widespread pain (*n* = 167)	1.01 (0.98–1.04)	**1.05** (**1.02–1.09)**	**1.06** (**1.02–1.10)**	1.00 (0.97–1.04)
Chronic fatigue (*n* = 204)	**1.06** (**1.03–1.09)**	**1.04** (**1.01–1.09)**	**1.07** (**1.03–1.11)**	0.99 (0.96–1.02)

Except where otherwise indicated, the table consists of logistic regression models with neuroticism, perceived stress, adverse life events and self-efficacy in the same model, further adjusted for baseline levels of gender, age and social status. Significant results (i.e. 95% confidence intervals that do not contain 1) are bolded. FSD, functional somatic disorder; FSS, functional somatic syndromes.

a. Unadjusted because the number of cases was too low.

b. Only adjusted for baseline values of perceived stress, general self-efficacy, neuroticism and accumulated number of adverse life events.

The same tendencies were seen for the FSD diagnoses established with the diagnostic interview (Table 3); however, only a minority of the associations were significant (i.e. with a 95% confidence interval that did not contain 1). The baseline accumulated number of ALEs predicted incident overall FSD (odds ratio 1.10, 95% CI 1.03–1.17) and incident multi-organ type FSD (odds ratio 1.18, 95% CI 1.06–1.31) at follow-up.

For the FSS, only baseline perceived stress predicted incident irritable bowel at follow-up (odds ratio 1.06, 95% CI 1.02–1.11), whereas both baseline perceived stress (odds ratio 1.05, 95% CI 1.02–1.09) and accumulated number of ALEs (odds ratio 1.06, 95% CI 1.02–1.10) predicted incident CWP at follow-up (Table 3). Chronic fatigue was predicted by baseline neuroticism (odds ratio 1.06, 95% CI 1.03–1.09), perceived stress (odds ratio 1.04, 95% CI 1.01–1.09) and accumulated number of ALEs (odds ratio 1.07, 95% CI 1.03–1.11).

### The role of changes in perceived stress, ALEs and self-efficacy for the development of incident FSDs

An increase between baseline and follow-up in perceived stress and number of ALEs were positively associated with the development of incident FSD at follow-up, whereas an increase in self-efficacy was a protective factor for incident FSD at follow-up (Table 4). For example, for overall FSD defined by symptom questionnaires, comparing two participants with equal baseline values on neuroticism, perceived stress, number of ALEs, self-efficacy, gender, age and social status but where one of them had a one-point larger increase in perceived stress between baseline and follow-up than the other, the participant had a 1.13 (95% CI 1.10–1.16) times higher odds of developing incident overall FSD at follow-up; a one-point difference in increased number of ALEs resulted in a 1.12 (95% CI 1.09–1.15) times higher odds of developing incident overall FSD at follow-up; and a one-point difference in increased self-efficacy resulted in a 0.96 (95% CI 0.94–0.99) times lower odds of developing incident overall FSD at follow-up.

**Table 4:** Odds of incident functional somatic disorders and functional somatic syndromes

	Difference in perceived stress; baseline to follow-up	Difference in adverse life events; baseline to follow-up	Difference in self-efficacy; baseline to follow-up
	Odds ratio (95% CI)	Odds ratio (95% CI)	Odds ratio (95% CI)
FSD defined by self-reported symptom questionnaires			
Overall FSD (*n* = 375)	**1.13** (**1.10–1.16)**	**1.12** (**1.04–1.21)**	**0.96** (**0.94–0.99)**
Single-organ FSD (*n* = 355)	**1.12** (**1.09–1.15)**	**1.12** (**1.04–1.21)**	**0.97** (**0.94–0.99)**
Multi-organ FSD (*n* = 20)^a^	**1.22** (**1.13–1.31)**	1.16 (0.89–1.53)	**0.89** (**0.82–0.97)**
FSD diagnosis established by means of diagnostic interviews			
Overall FSD (*n* = 59)^b^	1.06 (1.00–1.13)	0.90 (0.72–1.13)	0.94 (0.88–1.01)
Single-organ FSD (*n* = 50)^b^	1.04 (0.97–1.10)	0.97 (0.68–1.12)	0.94 (0.97–1.00)
Multi-organ FSD (*n* = 9)^a^	1.01 (0.89–1.15)	1.15 (0.76–1.75)	0.99 (0.86–1.13)
FSS defined by self-reported symptom questionnaires			
Irritable bowel (*n* = 108)	**1.08** (**1.04–1.12)**	0.97 (0.84–1.12)	0.98(0.94–1.02)
Chronic widespread pain (*n* = 167)	**1.06** (**1.02–1.09)**	1.07 (0.96–1.19)	0.97 (0.93–1.00)
Chronic fatigue (*n* = 204)	**1.17** (**1.13–1.21)**	**1.11** (**1.01–1.23)**	**0.91** (**0.88–0.95)**

The table consists of separate logistic regression models with each variable (difference in perceived stress, difference in number of adverse life events and difference in self-efficacy between baseline and follow-up) as the primary independent variable, adjusted for baseline levels of neuroticism, perceived stress, adverse life events and self-efficacy, as well as for gender, age and social status. Significant results (i.e. 95% confidence intervals that do not contain 1), are bolded. FSD, functional somatic disorder; FSS, functional somatic syndromes.

a. Unadjusted because the number of cases was too low.

b. Only adjusted for baseline values of perceived stress, general self-efficacy, neuroticism and accumulated number of adverse life events.

For FSD diagnoses established with the diagnostic interview, some of the same tendencies were seen for perceived stress and self-efficacy. However, the direction of associations was generally more inconsistent (Table 4).

For FSS, an increase in perceived stress from baseline to follow-up was positively associated with both incident irritable bowel and CWP, and particularly with chronic fatigue (odds ratio 1.17, 95% CI 1.13–1.21), at follow-up (Table 4). An increase in the accumulated number of ALEs was positively associated with incident chronic fatigue (odds ratio 1.11, 95% CI 1.01–1.23) at follow-up, whereas an increase in self-efficacy was negatively associated with incident chronic fatigue at follow-up (odds ratio 0.91, 95% CI 0.88–0.95).

## Discussion

In this population-based follow-up study, it was shown that higher baseline levels of perceived stress, neuroticism and accumulated number of ALEs predicted the development of new cases of FSD over a 5-year time period. In contrast, the baseline level of self-efficacy seemed to be of less importance. Furthermore, an increase in perceived stress between baseline and follow-up was consistently associated with new onset of FSD, and an increase in self-efficacy seemed to be a protective factor.

Our findings are in line with other studies on the subject. A recent systematic review by Kitselaar et al^31^ found that high neuroticism and self-discipline predicted onset of functional somatic symptoms, irritable bowel syndrome, fibromyalgia and chronic fatigue syndrome; stress predicted onset of irritable bowel syndrome and chronic back pain; and negative life events – especially childhood adversities – predicted onset of a variety of FSDs, such as functional somatic symptoms, medically unexplained symptoms, fibromyalgia, irritable bowel syndrome and chronic fatigue syndrome. The authors conclude that, overall, psychological factors are critical contributors to the onset of FSD. A general population-based study on the large Dutch LifeLines cohort has indicated that higher stress related to chronic ill health predicted onset of irritable bowel syndrome, fibromyalgia and chronic fatigue syndrome.^32^ Furthermore, the study showed some difference in predictors for specific FSD that do not fully comply with our results: Contrary to our findings that a higher accumulated number of ALEs predicted CWP and chronic fatigue, they found that high negative life event scores predicted irritable bowel syndrome and chronic fatigue syndrome, but not fibromyalgia. Furthermore, our study found neuroticism to predict chronic fatigue, whereas the LifeLines study found high neuroticism to predict fibromyalgia, but not chronic fatigue syndrome. However, the FSS diagnoses in the LifeLines study are based on participants’ self-report only, and they used other statistical methods for evaluating the possible predictors than our study. Hence, despite the impressive sample size (*N* = 153 180), their findings are not completely comparable to those of the present study.

Generally, the generalisability of the effect sizes found in the current study are hard to compare with what have been found in other studies on the same subject, because of heterogeneity of measures and statistical modelling. Also, it may be difficult to establish the clinical relevancy of the associations because information on cut-offs for clinically relevant differences are lacking. However, in the current study, we report odds ratios comparing individuals only differing one point on the measurements. Hence, even a small odds ratio would imply an important difference; for example, comparing two individuals differing by only one point on the NEO-PI-Rsf (ranging 0–48) at baseline, the individual with the highest score would have 16% higher odds of having developed multi-organ FSD at follow-up. For a difference on the NEO-PI-Rsf of five points, the individual scoring highest would more than double the odds (110%) of having developed multi-organ FSD at follow-up. It would be reasonable to conclude that this would be a difference with clinical relevance.

The interplay of these psychological factors and their relationship with FSD may be understood through a cognitive–behavioural model,^33^ and our findings (particularly concerning neuroticism) are equally in line with more recent predictive-processing models of FSD positing that negative affectivity is a vulnerability factor that relates to both psychopathology and somatic symptoms.^34,35^ Individuals with high neuroticism may have a heightened reactivity to stressors, and a tendency to respond with negative emotions and physical arousal to external distressing events such as negative life events and traumas.^33^ The cognitive appraisal of stressful situations or the experience of ALEs is also influenced by the level of self-efficacy, which correlates negatively with neuroticism.^33^ The influence of an individual's level of self-efficacy/coping mechanisms may also explain why a mismatch between, for example, ‘objective stressful events’ and self-perceived stress occur: an individual may have experienced various stressful ALEs without reporting higher levels of perceived stress. Through a normal life course, external events are continually perceived and determined in the brain as threatening or non-threatening through an individual's behavioural and psychological responses. Optimally, this would create adaptive processes that promote survival (i.e. allostasis). Several biological factors also influence these processes (e.g. hormones such as cortisol and adrenalin, the autonomic nervous system, and pro- and anti-inflammatory cytokines). However, when dysregulated, these mediators may lead to allostatic load and give rise to pathology.^36^

The present study focused on the accumulated number of lifetime ALEs, but previous research has indicated that in particular childhood adversities may be of particular interest for the development and prognosis of FSD. Population-based studies have shown childhood trauma to predict irritable bowel syndrome,^37,38^ fibromyalgia,^39^ chronic back pain^40–42^ and chronic fatigue syndrome.^43^ To gain further knowledge on how to prevent, diagnose and treat FSD, future studies may preferably investigate the role of childhood adversities and FSD.

In the current study, perceived stress in particular was a predictor for new onset of FSD. Likewise, an increase in perceived stress was associated with incident FSD. In a previous study, we found perceived stress to be strongly associated with FSD when comparing individuals without FSD and individuals with severe physical disease.^11^ However, because of its cross-sectional design, it was not possible to determine whether high perceived stress was a risk factor for developing FSD or a consequence of having FSD. The current study takes this discussion a step further and makes a valid argument that perceived stress is a risk factor for the onset of FSD.

### Strengths and limitations

The results from the present population-based study should be interpreted in the light of some strengths and limitations.

The inclusion of a large, randomly obtained, population-based sample with an almost equal distribution of the two genders constitutes an important strength, together with the inclusion of several definitions of FSD as well as the self-reported questionnaires and diagnostic interviews for assessing FSD.

However, the response rates of 29.5% for the baseline cohort and 58.8% for the 5-year follow-up investigation may have resulted in a risk of selection bias. For the baseline investigation, a non-responder analysis has shown that selection bias did not seem to noticeably influence the social parameters.^44^ However, the non-responder analysis for the 5-year follow-up investigation from the current study showed that the non-responders had a higher proportion of FSD than responders. Likewise, non-responders had slightly higher baseline levels of perceived stress and neuroticism, and lower number of ALEs. Hence, the non-response for the 5-year follow-up investigation might have biased the results of the current study toward an underestimation of the associations between the psychological factors and FSD.

The study included two measurement points of FSD, perceived stress, self-efficacy and number of ALEs, and only one measurement point of neuroticism. It was therefore not possible to investigate if a change in neuroticism between baseline and follow-up was associated with incident FSD at follow-up. Furthermore, with this study design it was only possible to draw a conclusion about cross-sectional associations with change in scores of perceived stress, self-efficacy, and number of ALEs and FSD at follow-up. It was not possible to establish how a change in scores would affect a definite onset and prognosis of FSD. To do so, three measurement points for FSD would be needed.

Some limitations of the statistical analyses must also be addressed. The number of incident cases was low for some of the FSD categories, especially for multi-organ FSD and the interview-diagnosed cases in general. Regarding these cases, it was therefore not possible to run multiple variable analyses and adjust for all relevant confounders. The estimates obtained from these analyses may therefore be less confident. Furthermore, all statistical models assumed that there were no interactions between the independent variables. Therefore, to emphasise the results from the current study, similar analyses should preferably be replicated in future studies.

## Supporting information

Petersen et al. supplementary material Petersen et al. supplementary material

## Data Availability

The data that support the findings of this study are available from the corresponding author, M.W.P, upon reasonable request.
