# Supplementary material for: Neuroticism, perceived stress, adverse life events and self-efficacy as predictors of the development of functional somatic disorders: longitudinal population-based study (DanFunD)
Source: BJPsych Open. 2024 Jan 25;10(1):e34. doi: 10.1192/bjo.2023.644 (PMC10897700; doi:10.1192/bjo.2023.644)
Supplement: Petersen et al. supplementary material [file S2056472423006440sup001.docx]

Supplemental Materials

**Fig S1: Methodological overview of the study design**

**
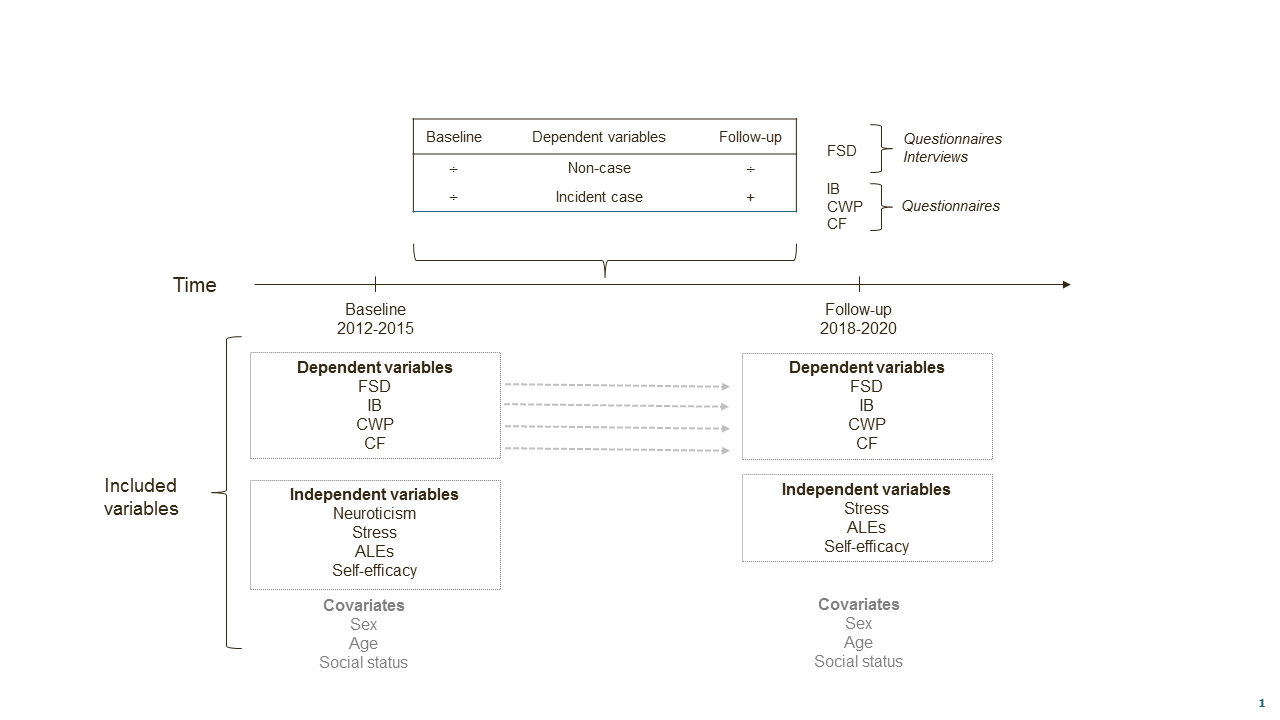
**

Abbreviations: FSD=functional somatic disorders; IB=irritable bowel; CWP=chronic widespread pain; CF=chronic fatigue; stress=perceived stress; ALEs=accumulated number of adverse life events.

**Fig S2: Flow of study participants**


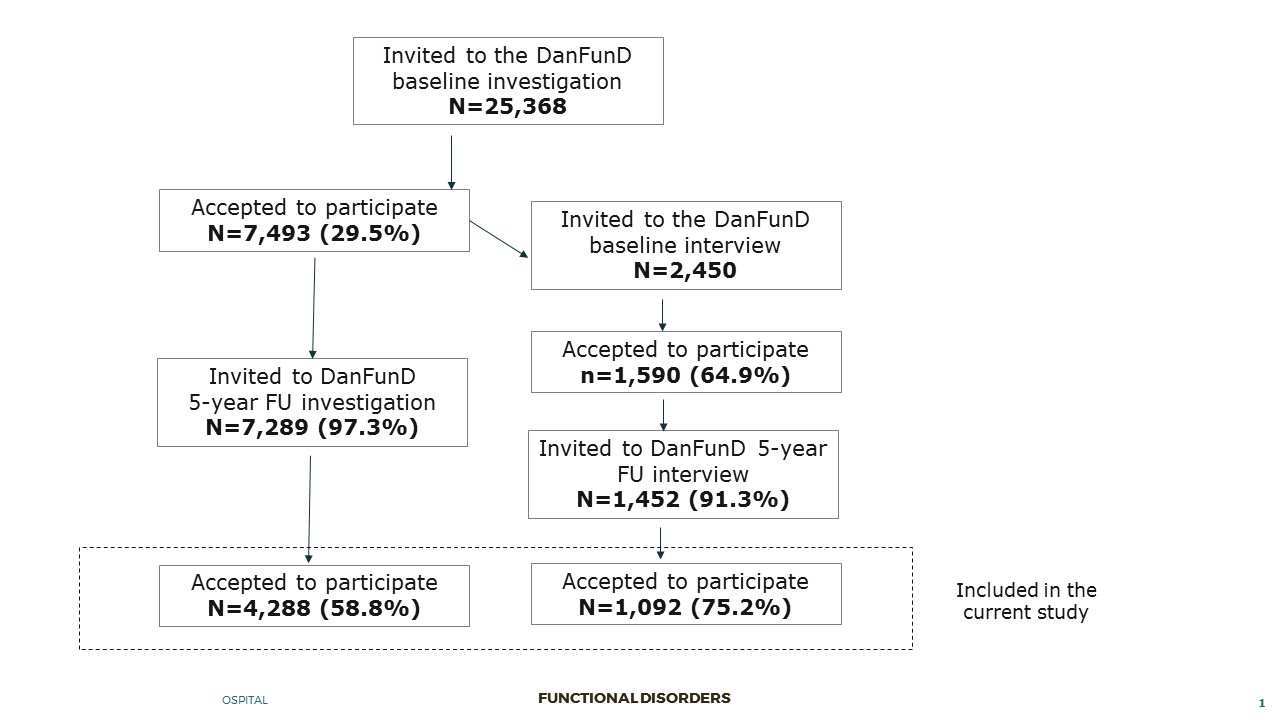


Abbreviations: FU = follow-up.
